# Supplementary material for: Effectiveness of integrating cervical cancer prevention strategies into HIV care programmes: A mixed-methods systematic review protocol
Source: PLoS One. 2025 May 19;20(5):e0306496. doi: 10.1371/journal.pone.0306496 (PMC12088019; doi:10.1371/journal.pone.0306496)
Supplement: S1 Appendix — (DOCX) [file pone.0306496.s002.docx]

**S1 Appendix. Database literature search strategy.**

Table 1: EBSCO Host (Academic Search Premier, Africa-Wide Information, Cumulative Index to Nursing and Allied Health Literature [CINAHL], Health Source - Consumer Edition, Health Source: Nursing/Academic Edition, APA PsycArticles, APA PsycInfo) literature search strategy.

| **Search** | **Query** | **Items found** |
| --- | --- | --- |
| #1 | Adolescent OR youth OR women OR “young women” OR female OR “young female” OR “young girl” OR “young female adolescent” |  |
| #2 | “human immunodeficiency virus” OR “human immuno deficiency virus” OR “human immune deficiency virus” OR hiv OR “hiv positive” OR “hiv infection*” OR “hiv aids” OR “living with hiv” OR “living with human immunodeficiency virus” |  |
| #3 | #1 AND #2 |  |
| #4 | “hpv vaccin*” OR “hpv immune*” OR “human papillomavirus vaccin*” OR “human papillomavirus immun*” OR “human papilloma virus vaccin*” OR “human papilloma virus immun*” OR “cervical cancer vaccin*” OR “cervical cancer immun*” |  |
| #5 | “cervical cancer screening” OR “early detection of cervical cancer” OR “hpv screening” OR “human papilloma virus screening” OR “human papillomavirus screening” OR “conventional cytology test*” OR “pap smear” OR “pap test” OR “pap smear based screening” OR “pap smear exam*” OR “pap smear screening*” OR “cervical smear” OR “vaginal smear” OR “papanicolaou smear” OR “papanicolaou smear screening” OR “papanicolaou smear test*” OR “cervical smear test” OR “papanicolaou test*” OR “cervical cytology” OR “cytology cervical smears” OR “papanicolaou stained smears” OR “liquid based cytology*” OR lbc OR “human papillomavirus dna test*” OR “human papilloma virus dna test*” OR “hpv dna test*” OR “human papillomavirus dna screening” OR “hpv dna screening” OR “human papilloma virus test*” OR “human papillomavirus test*” OR “hpv dna based tests” OR “hpv dna based testing” OR “human papilloma virus dna detection” OR “human papillomavirus dna detection” OR “visual inspection screening” OR “visual inspection of cervix*” OR “visual inspection with acetic*” OR “visual inspection with acetic acid via” OR “visual inspection with lugo*” OR “visual inspection with lugol s iodine vili” |  |
| #6 | “cervical cancer treatment” OR “hpv treatment” OR “human papillomavirus treatment” OR “hpv therapy” OR “cervical cancer therapy” OR “cervical cancer therapeutics” OR ablation OR “pre treatment” OR excision OR conization OR cryotherapy OR leep OR “loop electrosurgical excision*” OR “cone biopsy” OR laser* OR “cold coagulation” OR “large loop excision of transformation zone” OR lletz OR colposcopy |  |
| #7 | “cervical cancer education*” OR “hpv education” OR “human papillomavirus education” OR “cervical cancer knowledge*” OR “hpv knowledge*” OR “human papillomavirus knowledge” OR “human papilloma virus knowledge” OR “cervical cancer awareness” OR “hpv awareness” OR “human papillomavirus awareness” OR willingness |  |
| #8 | #4 OR #5 OR #6 OR #7 |  |
| #9 | “hiv care” OR “hiv services” OR “hiv clinics” OR “hiv centre*” OR “hiv healthcare*” OR clinic OR hospital OR “public health clinic*” OR “public health hospital*” OR “private healthcare facilities” OR “private healthcare facility” OR “public health facility” OR “public health facilities” OR “community based organisation*” OR “family planning clinic” OR “womens health clinic” OR “antenatal clinic” OR “maternity clinic” OR “outreach assessment clinics” OR “mobile clinics” OR “community based organization” OR “medical centre*” OR “primary healthcare facility” OR “primary healthcare clinic*” |  |
| #10 | #3 AND #8 AND #9 |  |
| Filters | Full text |  |
|  | Academic journals |  |
|  | Females |  |

Table 2: Cochrane Central Library literature search strategy.

| **Search** | **Query** | **Items found (TRIALS)** |
| --- | --- | --- |
| #1 | (Adolescent OR youth OR women OR “young women” OR female OR “young female” OR “young girl” OR “young female adolescent”):ti,ab,kw |  |
| #2 | (“human immunodeficiency virus” OR “human immuno deficiency virus” OR “human immune deficiency virus” OR hiv OR “hiv positive” OR hiv NEXT infection* OR “hiv aids” OR “living with hiv” OR “living with human immunodeficiency virus”):ti,ab,kw |  |
| #3 | #1 AND #2 |  |
| #4 | (hpv NEXT vaccin* OR hpv NEXT immune* OR human papillomavirus NEXT vaccin* OR human papillomavirus NEXT immun* OR human papilloma virus NEXT vaccin* OR human papilloma virus NEXT immun* OR cervical cancer NEXT vaccin* OR cervical cancer NEXT immun*):ti,ab,kw |  |
| #5 | (“cervical cancer screening” OR “early detection of cervical cancer” OR “hpv screening” OR “human papilloma virus screening” OR “human papillomavirus screening” OR conventional cytology NEXT test* OR “pap smear” OR “pap test” OR “pap smear based screening” OR pap smear NEXT exam* OR pap smear NEXT screening* OR “cervical smear” OR “vaginal smear” OR “papanicolaou smear” OR “papanicolaou smear screening” OR papanicolaou smear NEXT test* OR “cervical smear test” OR papanicolaou NEXT test* OR “cervical cytology” OR “cytology cervical smears” OR “papanicolaou stained smears” OR liquid based NEXT cytology* OR lbc OR human papillomavirus dna NEXT test* OR human papilloma virus dna NEXT test* OR hpv dna NEXT test* OR “human papillomavirus dna screening” OR “hpv dna screening” OR human papilloma virus NEXT test* OR human papillomavirus NEXT test* OR “hpv dna based tests” OR “hpv dna based testing” OR “human papilloma virus dna detection” OR “human papillomavirus dna detection” OR “visual inspection screening” OR visual inspection of NEXT cervix* OR visual inspection with NEXT acetic* OR “visual inspection with acetic acid via” OR visual inspection with NEXT lugo* OR “visual inspection with lugol s iodine vili”):ti,ab,kw |  |
| #6 | ("cervical cancer treatment" OR "cervical cancer therapy" OR "precancerous lesions treatment" OR "hpv treatment" OR "human papillomavirus treatment" OR "hpv therapy"):ti,ab,kw |  |
| #7 | (cervical cancer NEXT education* OR “hpv education” OR “human papillomavirus education” OR cervical cancer NEXT knowledge* OR hpv NEXT knowledge* OR “human papillomavirus knowledge” OR “human papilloma virus knowledge” OR “cervical cancer awareness” OR “hpv awareness” OR “human papillomavirus awareness” OR willingness):ti,ab,kw |  |
| #8 | #4 OR #5 OR #6 OR #7 |  |
| #9 | ("hiv care" OR "hiv services" OR "hiv clinics" OR "hiv centre" OR "hiv center" OR "hiv healthcare"):ti,ab,kw |  |
| #10 | #3 AND #8 AND #9 |  |

Table 3: PubMed literature search strategy.

| **Search** | **Query** | **Items found** |
| --- | --- | --- |
| #1 | Adolescent [Title/Abstract] OR youth [Title/Abstract] OR women [Title/Abstract] OR young women [Title/Abstract] OR female [Title/Abstract] OR young female [Title/Abstract] OR young girl [Title/Abstract] OR young female adolescent [Title/Abstract] |  |
| #2 | human immunodeficiency virus [Title/Abstract] OR human immuno deficiency virus [Title/Abstract] OR human immune deficiency virus [Title/Abstract] OR hiv [Title/Abstract] OR hiv positive [Title/Abstract] OR hiv infection* [Title/Abstract] OR hiv aids [Title/Abstract] OR living with hiv [Title/Abstract] OR living with human immunodeficiency virus [Title/Abstract] |  |
| #3 | #1 AND #2 |  |
| #4 | hpv vaccin* [Title/Abstract] OR hpv immune* [Title/Abstract] OR human papillomavirus vaccin* [Title/Abstract] OR human papillomavirus immun* [Title/Abstract] OR human papilloma virus vaccin* [Title/Abstract] OR human papilloma virus immun* [Title/Abstract] OR cervical cancer vaccin* [Title/Abstract] OR cervical cancer immun* [Title/Abstract] |  |
| #5 | cervical cancer screening [Title/Abstract] OR early detection of cervical cancer [Title/Abstract] OR hpv screening [Title/Abstract] OR human papilloma virus screening [Title/Abstract] OR human papillomavirus screening [Title/Abstract] OR conventional cytology test* [Title/Abstract] OR pap smear [Title/Abstract] OR pap test [Title/Abstract] OR pap smear based screening [Title/Abstract] OR pap smear exam* [Title/Abstract] OR pap smear screening* [Title/Abstract] OR cervical smear [Title/Abstract] OR vaginal smear [Title/Abstract] OR papanicolaou smear [Title/Abstract] OR papanicolaou smear screening [Title/Abstract] OR papanicolaou smear test* [Title/Abstract] OR cervical smear test [Title/Abstract] OR papanicolaou test* [Title/Abstract] OR cervical cytology [Title/Abstract] OR cytology cervical smears [Title/Abstract] OR papanicolaou stained smears [Title/Abstract] OR liquid based cytology* [Title/Abstract] OR lbc [Title/Abstract] OR human papillomavirus dna test* [Title/Abstract] OR human papilloma virus dna test* [Title/Abstract] OR hpv dna test* [Title/Abstract] OR human papillomavirus dna screening [Title/Abstract] OR hpv dna screening [Title/Abstract] OR human papilloma virus test* [Title/Abstract] OR human papillomavirus test* [Title/Abstract] OR hpv dna based tests [Title/Abstract] OR hpv dna based testing [Title/Abstract] OR human papilloma virus dna detection [Title/Abstract] OR human papillomavirus dna detection [Title/Abstract] OR visual inspection screening [Title/Abstract] OR visual inspection of cervix* [Title/Abstract] OR visual inspection with acetic* [Title/Abstract] OR visual inspection with acetic acid via [Title/Abstract] OR visual inspection with lugo* [Title/Abstract] OR visual inspection with lugol s iodine vili [Title/Abstract] |  |
| #6 | cervical cancer treatment [Title/Abstract] OR hpv treatment [Title/Abstract] OR human papillomavirus treatment [Title/Abstract] OR hpv therapy [Title/Abstract] OR cervical cancer therapy [Title/Abstract] OR cervical cancer therapeutics [Title/Abstract] OR ablation [Title/Abstract] OR pre treatment [Title/Abstract] OR excision [Title/Abstract] OR conization [Title/Abstract] OR cryotherapy [Title/Abstract] OR leep [Title/Abstract] OR loop electrosurgical excision* [Title/Abstract] OR cone biopsy [Title/Abstract] OR laser* [Title/Abstract] OR cold coagulation [Title/Abstract] OR large loop excision of transformation zone [Title/Abstract] OR lletz [Title/Abstract] OR colposcopy [Title/Abstract] |  |
| #7 | cervical cancer education* [Title/Abstract] OR hpv education [Title/Abstract] OR human papillomavirus education [Title/Abstract] OR cervical cancer knowledge* [Title/Abstract] OR hpv knowledge* [Title/Abstract] OR human papillomavirus knowledge [Title/Abstract] OR human papilloma virus knowledge [Title/Abstract] OR cervical cancer awareness [Title/Abstract] OR hpv awareness [Title/Abstract] OR human papillomavirus awareness [Title/Abstract] OR willingness [Title/Abstract] |  |
| #8 | #4 OR #5 OR #6 OR #7 |  |
| #9 | hiv care [Title/Abstract] OR hiv services [Title/Abstract] OR hiv clinics [Title/Abstract] OR hiv centre* [Title/Abstract] OR hiv healthcare* [Title/Abstract] OR clinic [Title/Abstract] OR hospital [Title/Abstract] OR public health clinic* [Title/Abstract] OR public health hospital* [Title/Abstract] OR private healthcare facilities [Title/Abstract] OR private healthcare facility [Title/Abstract] OR public health facility [Title/Abstract] OR public health facilities [Title/Abstract] OR community based organisation* [Title/Abstract] OR family planning clinic [Title/Abstract] OR womens health clinic [Title/Abstract] OR antenatal clinic [Title/Abstract] OR maternity clinic [Title/Abstract] OR outreach assessment clinics [Title/Abstract] OR mobile clinics [Title/Abstract] OR community based organization [Title/Abstract] OR medical centre* [Title/Abstract] OR primary healthcare facility [Title/Abstract] OR primary healthcare clinic* [Title/Abstract] |  |
| #10 | #3 AND #8 AND #9 |  |
| Filters | Abstract, Full text, Female |  |

Table 4: Scopus literature search strategy.

| **Search** | **Query** | **Items found** |
| --- | --- | --- |
| #1 | TITLE-ABS-KEY ( adolescent OR youth OR women OR "young women" OR female OR "young female" OR "young girl" OR "young female adolescent" ) |  |
| #2 | TITLE-ABS-KEY ( "human immunodeficiency virus" OR "human immuno deficiency virus" OR "human immune deficiency virus" OR hiv OR "hiv positive" OR "hiv infection*" OR "hiv aids" OR "living with hiv" OR "living with human immunodeficiency virus" ) |  |
| #3 | #1 AND #2 |  |
| #4 | TITLE-ABS-KEY ( "hpv vaccin*" OR "hpv immune*" OR "human papillomavirus vaccin*" OR "human papillomavirus immun*" OR "human papilloma virus vaccin*" OR "human papilloma virus immun*" OR "cervical cancer vaccin*" OR "cervical cancer immun*" ) |  |
| #5 | TITLE-ABS-KEY ( "cervical cancer screening" OR "early detection of cervical cancer" OR "hpv screening" OR "human papilloma virus screening" OR "human papillomavirus screening" OR "conventional cytology test*" OR "pap smear" OR "pap test" OR "pap smear based screening" OR "pap smear exam*" OR "pap smear screening*" OR "cervical smear" OR "vaginal smear" OR "papanicolaou smear" OR "papanicolaou smear screening" OR "papanicolaou smear test*" OR "cervical smear test" OR "papanicolaou test*" OR "cervical cytology" OR "cytology cervical smears" OR "papanicolaou stained smears" OR "liquid based cytology*" OR lbc OR "human papillomavirus dna test*" OR "human papilloma virus dna test*" OR "hpv dna test*" OR "human papillomavirus dna screening" OR "hpv dna screening" OR "human papilloma virus test*" OR "human papillomavirus test*" OR "hpv dna based tests" OR "hpv dna based testing" OR "human papilloma virus dna detection" OR "human papillomavirus dna detection" OR "visual inspection screening" OR "visual inspection of cervix*" OR "visual inspection with acetic*" OR "visual inspection with acetic acid via" OR "visual inspection with lugo*" OR "visual inspection with lugol s iodine vili" ) |  |
| #6 | TITLE-ABS-KEY ( "cervical cancer treatment" OR "cervical cancer therapy" OR "precancerous lesions treatment" OR "hpv treatment" OR "human papillomavirus treatment" OR "hpv therapy" ) |  |
| #7 | TITLE-ABS-KEY ( "cervical cancer education*" OR "hpv education" OR "human papillomavirus education" OR "cervical cancer knowledge*" OR "hpv knowledge*" OR "human papillomavirus knowledge" OR "human papilloma virus knowledge" OR "cervical cancer awareness" OR "hpv awareness" OR "human papillomavirus awareness" OR willingness ) |  |
| #8 | #4 OR #5 OR #6 OR #7 |  |
| #9 | TITLE-ABS-KEY ( "hiv care" OR "hiv services" OR "hiv clinics" OR "hiv centre" OR "hiv center" OR "hiv healthcare" ) |  |
| #10 | #3 AND #8 AND #9 |  |
| #11 | Filters: Articles |  |

Table 5: Web of Science literature search strategy.

| **Search** | **Query** | **Items found** |
| --- | --- | --- |
| #1 | TS=(Adolescent OR youth OR women OR “young women” OR female OR “young female” OR “young girl” OR “young female adolescent”) |  |
| #2 | TS=(“human immunodeficiency virus” OR “human immuno deficiency virus” OR “human immune deficiency virus” OR hiv OR “hiv positive” OR “hiv infection*” OR “hiv aids” OR “living with hiv” OR “living with human immunodeficiency virus”) |  |
| #3 | #1 AND #2 |  |
| #4 | TS=(“hpv vaccin*” OR “hpv immune*” OR “human papillomavirus vaccin*” OR “human papillomavirus immun*” OR “human papilloma virus vaccin*” OR “human papilloma virus immun*” OR “cervical cancer vaccin*” OR “cervical cancer immun*” ) |  |
| #5 | TS=(“cervical cancer screening” OR “early detection of cervical cancer” OR “hpv screening” OR “human papilloma virus screening” OR “human papillomavirus screening” OR “conventional cytology test*” OR “pap smear” OR “pap test” OR “pap smear based screening” OR “pap smear exam*” OR “pap smear screening*” OR “cervical smear” OR “vaginal smear” OR “papanicolaou smear” OR “papanicolaou smear screening” OR “papanicolaou smear test*” OR “cervical smear test” OR “papanicolaou test*” OR “cervical cytology” OR “cytology cervical smears” OR “papanicolaou stained smears” OR “liquid based cytology*” OR lbc OR “human papillomavirus dna test*” OR “human papilloma virus dna test*” OR “hpv dna test*” OR “human papillomavirus dna screening” OR “hpv dna screening” OR “human papilloma virus test*” OR “human papillomavirus test*” OR “hpv dna based tests” OR “hpv dna based testing” OR “human papilloma virus dna detection” OR “human papillomavirus dna detection” OR “visual inspection screening” OR “visual inspection of cervix*” OR “visual inspection with acetic*” OR “visual inspection with acetic acid via” OR “visual inspection with lugo*” OR “visual inspection with lugol s iodine vili”) |  |
| #6 | TS=(“cervical cancer treatment” OR “cervical cancer therapy” OR “precancerous lesions treatment” OR “hpv treatment” OR “human papillomavirus treatment” OR “hpv therapy”) |  |
| #7 | TS=(“cervical cancer education*” OR “hpv education” OR “human papillomavirus education” OR “cervical cancer knowledge*” OR “hpv knowledge*” OR “human papillomavirus knowledge” OR “human papilloma virus knowledge” OR “cervical cancer awareness” OR “hpv awareness” OR “human papillomavirus awareness” OR willingness) |  |
| #8 | #4 OR #5 OR #6 OR #7 |  |
| #9 | TS=(“hiv care” OR “hiv services” OR “hiv centre” OR “hiv center” OR “hiv clinics” OR “hiv healthcare”) |  |
| #10 | #3 AND #8 AND #9 |  |
| Filters | Document types: "Article" and "Early Access" |  |

Table 6: Google Scholar literature search strategy.

| **Search** | **Query** | **Items found** |
| --- | --- | --- |
| #1 | "adolescent girls and young women" AND "living with HIV" AND ("cervical cancer screening" OR "HPV vaccination" OR cervical cancer treatment") AND "HIV services" AND “integration” |  |
