# Supplementary material for: Effectiveness of integrating cervical cancer prevention strategies into HIV care programmes: A mixed-methods systematic review protocol
Source: PLoS One. 2025 May 19;20(5):e0306496. doi: 10.1371/journal.pone.0306496 (PMC12088019; doi:10.1371/journal.pone.0306496)
Supplement: Attachment — Submitted filename: Response to Reviewers.docx [file pone.0306496.s005.docx]

**Response to Reviewer 1**

**Comment 1:**

This systemic review protocol appears too ambitious in attempting to include all cervical cancer prevention strategies. Cervical cancer prevention encompasses primary prevention: HPV vaccination, and secondary prevention: various screening methods. Attempting to address all these strategies simultaneously within one systemic review could result in a lack of focus and clarity. Suggest narrow down to just one strategy.

**Author’s response to comment 1:**

We thank the reviewer for this insightful comment on the scope of this systematic review protocol. Since submitting this manuscript for peer review in June 2024, we have completed the literature search and screening and are pleased to share that we identified a total of 30 eligible articles.

While we acknowledge the challenges of addressing all cervical cancer prevention strategies in one systematic review, there are compelling reasons to maintain this comprehensive approach. First, the scope of this systematic review is foregrounded on the 90–70–90 targets set as part of the global strategy to accelerate the elimination of cervical cancer as a public health problem, with a focus on primary and secondary prevention among adolescent girls and young women (AGYW) living with HIV. Whilst existing literature focuses on individual prevention strategies particularly for women in the general population, there is currently none that provides a holistic approach that encompasses all prevention strategies among AGYW living with HIV. It is this critical evidence gap that we aim to bridge towards enhancing evidence-based decision-making and practice where scaling-up elimination of cervical cancer as a public health threat for AGYW living with HIV is concerned. Further to this, we are specifically interested in exploring integration of these prevention strategies as part of HIV care services (i.e. if and how these services are being integrated as part of HIV care pathways and the influence of integration on service uptake and utilisation). We anticipate that our findings could provide insights into the global stance on integration strategies. Our review will further highlight gaps in terms of which prevention strategies are underutilised. Highlighting these gaps would be crucial in informing policy decisions on which prevention strategies require further development and planning. Limiting this review to just one strategy risks missing these crucial insights.

**Comment 2:**

It is recommended that the authors adopt the PICO framework to effectively structure this systematic review. Firstly, the target population needs to be clearly defined. The authors should provide a rationale for selecting women aged 9-25 years, particularly given that screening methods is typically aimed at women aged 25-60.

**Author’s response to comment 2:**

We thank the reviewer for this comment as it highlighted the fact that the rationale outlined in the protocol lacked adequate justification for the specific elements of the PICO framework adopted for this review. We have now revised the rationale for this protocol with the guidance provided which can be found on page 9-13, line 193-269, and reads as follows:

*“Rationale for the Review*

*Guided by the PICO (population, intervention, comparator and outcome) framework [17,18] the specific details of the population, intervention, comparator and outcomes for this review are outlined in Table 1.*

*Table 1. Summary of the target population, interventions, comparator and outcomes (PICO) set out for this review*

| ***PICO framework*** | ***Description*** |
| --- | --- |
| ***Population (P)*** | *Adolescent girls and young women (AGYW) living with HIV (9-25 years)* |
| ***Interventions (I)*** | *Integration of cervical cancer prevention strategies with HIV care*  *Primary prevention:*   1. *HPV vaccination* 2. *Educational interventions*   *Secondary prevention:*   1. *Cervical cancer screening* 2. *Treatment of precancerous lesions.* |
| ***Comparator (C)*** | *Where studies are available that make use of before and after designs, we will explore the effectiveness of intervention versus no intervention or integration versus no integration.* |
| ***Outcomes (O)*** | 1. *A description of models used to integrate cervical cancer prevention strategies with existing HIV care programs will be identified and described.* 2. *The effectiveness of integrating cervical cancer prevention strategies into existing HIV care programs will be assessed in terms of the following outcomes:*    2.1 *The uptake of cervical cancer prevention strategies (HPV vaccination, cervical cancer screening, treatment of precancerous lesions, and educational interventions) by AGYW living with HIV following integration*    2.2 *The knowledge, awareness, and willingness of AGYW living with HIV to utilise and adhere to cervical cancer prevention strategies following integration.*   *In addition, this review would also report qualitative data relating to the*  *knowledge, awareness and willingness of AGYW living with HIV to*  *utilise and adhere to cervical cancer prevention strategies following*  *integration.* |

*Population:*

*Focusing this review on AGYW (9-25 years) living with HIV is pertinent, given the distinct factors that heighten their lifetime vulnerability to cervical cancer and position them to gain significant benefits from integrated prevention strategies (Table 1). AGYW living with HIV are at a significantly higher risk of persistent HPV infection, leading to a more rapid progression to cervical precancer and potentially invasive cervical cancer [3]. This elevated risk necessitates early and sustained cervical cancer prevention efforts tailored specifically for this population to prevent early complications and premature death. Improving the uptake of these services in this age group would promote early prevention of cervical cancer with HPV vaccination and early detection or screening services thus ensuring significant long-term reduction in the incidence and mortality associated with cervical cancer.*

*Intervention:*

*HPV vaccination is the most effective strategy for preventing cervical cancer. HPV vaccines are highly effective at preventing HPV infections with an efficacy rate of 95% [1,5]. Furthermore, HPV vaccines are most effective if administered prior to HPV-exposure and therefore primarily targeted to adolescent girls (9-14 years of age), prior to sexual debut [7]. Prioritising the uptake of HPV vaccination among AGYW living with HIV would contribute significantly towards reducing the global burden of cervical cancer globally. At present the WHO recommends initiating cervical cancer screening among females living with HIV at the age of 25 years as part of secondary prevention strategies [6]. Given that AGYW are a high-risk population [3], screening among this population would promote early detection and treatment of pre-cancerous lesions, thus reducing the likelihood of developing invasive cervical cancer later in life. Lastly, adolescence and young adulthood are generally regarded as the critical period for developing lifelong health behaviours. Increasing the uptake of educational interventions about HPV infection and cervical cancer among AGYW living with HIV could present an opportunity to increase knowledge and promote early adoption of cervical cancer prevention strategies among this population.*

*Comparator:*

*The evidence base on cervical cancer prevention among AGYW and adult women within the general population is substantial and as such, this population will not be the focus of this review [19]. Given our specific interest in integrated services for AGYW living with HIV, we will explore utilisation of cervical cancer prevention services when integrated into HIV care pathways compared to no integration (Table 1).*

*Outcomes:*

*The co-occurrence of cervical cancer and HIV represents a significant public health challenge and requires more effort to be placed on improving delivery and uptake of current cervical cancer prevention and control measures. Cervical cancer prevention strategies should encompass multidisciplinary approaches that include HPV vaccination, screening, treatment of precancerous lesions, and educational interventions (Table 1). Utilising existing HIV care programmes as a means of integrating cervical cancer prevention strategies into routine HIV care provides an effective way for improving cervical cancer prevention for the most at-risk population and should be prioritised. As mentioned, existing literature primarily focuses on isolated aspects of either HIV care or cervical cancer prevention, or some but not all cervical cancer preventative strategies, thus leaving a fragmented understanding of the holistic benefits of integrating services for these public health concerns. Previous studies have considered integration of these programs [12], although, more focus is placed on cervical cancer screening as a prevention strategy as opposed to a comprehensive approach that also includes vaccination, education, and treatment of precancerous lesions. Moreover, the search end dates of previous reviews are outdated - being more than six years old - and do not consider recent recommendations and advancements in the field [12].* *This review aims to provide a comprehensive assessment of the effectiveness of, and knowledge, awareness, and willingness among AGYW living with HIV to utilise, integrated cervical cancer prevention strategies. The holistic approach intended for this review could provide further insights into the global stance on integration strategies. In doing this, gaps in terms of which strategies are underutilised, and less or more effective can be highlighted. Highlighting these gaps would be crucial in informing policy decisions on which prevention strategies require further development and planning. Limiting this review to just one strategy risks missing these crucial insights.*

*A mixed methods systematic review is an ideal research design for this study as it allows for an overall examination of both quantitative and qualitative data. The quantitative evidence will provide insights into the types of integration strategies adopted and their effectiveness, whilst the qualitative evidence can shed light on the knowledge, awareness, and willingness of AGYW living with HIV to utilise cervical cancer prevention strategies following integration. Findings from this review will have the potential to inform reforms to current policy and practice, thereby contributing to improving health outcomes for AGYW living with HIV.”*

**Comment 3:**

Regarding the intervention or exposure focus, which is central to this study protocol, it should be clarified whether the primary emphasis is on HPV vaccination as a method of primary prevention or on screening methods. Including both aspects may lead to confusion among readers.

**Author’s response to comment 3:**

We thank the reviewer for raising this concern. As indicated previously in the authors response to comment 1, this systematic review is aimed at gaining a holistic understanding of the effectiveness of integrating cervical cancer prevention strategies (i.e., both primary and secondary prevention strategies) within HIV care, particularly targeted towards AGYW living with HIV. With guidance from both reviewers, we have carefully re-structured this protocol to explicitly reflect this and systematically outline the outcomes of interest and how we intent sourcing and reporting such findings.

**Comment 4:**

Additionally, the study protocol should specify the comparison or control group more explicitly.

**Author’s response to comment 4:**

We have included further details and a table in the “*Rational for the review*” section (page 9-13, line 193-269) that outlines the specific details of the comparator and how it relates to the rest of the PICO dimensions set out for this review.

**Comment 5:**

The outcomes outlined in the study protocol are currently unclear and require further elaboration.

**Author’s response to comment 5:**

This comment is well noted, and we agree that further clarification and elaboration of the outcomes are necessary to ensure the protocol’s transparency and scientific rigor. We have now elaborated on the anticipated outcomes of this review for better clarity. A revised version can be found on page 16-17, line 338-376, which reads as follows:

“***Outcomes of interest***

1. *We will identify and describe types or models of integrated services utilised by AGYW living with HIV to access cervical cancer prevention services. Sigfrid et al, 2017 identify three models for integrating cervical cancer with HIV healthcare services [13]. The first model refers to within-clinic integration using internal staff, where the existing clinic structure and staff are used to incorporate a new set of services to complement services that are already provided [13]. In the second model, integration is achieved through co-location, where HIV and cervical cancer services are provided to the patient through coordination of care between different specialists or clinics within the same health care facility [13]. The third model involves complex programs of integration and coordination, including programs that integrated services by involving a range of different types of health workers (often from community health workers to clinical specialists) and facilities, and established systems to ensure clinical coordination and follow-up of patients [13]. Drawing on these three models, we will report on how cervical cancer prevention strategies are commonly integrated into HIV care services across the evidence base.*
2. *We will also report on the effectiveness of integrating cervical cancer prevention strategies into existing HIV care programs. This will be assessed in terms of the following outcomes:*
   2.1 *The uptake of cervical cancer prevention services (HPV vaccination, cervical cancer screening, treatment of precancerous lesions, and educational interventions) by AGYW living with HIV before versus after integration. Changes in measurable indicators related to the uptake of cervical cancer prevention strategies reported in included studies will be used to assess effectiveness. This can be numeric results (percentage or proportion), or measures of association related to uptake that have been reported before and after integration or those that compare uptake of services between integrated and non-integrated settings.*
   2.2 *The knowledge, awareness, and willingness of AGYW living with HIV to utilise and adhere to cervical cancer prevention strategies following integration. Changes in measurable indicators related to knowledge, awareness and willingness reported in included studies will be used to assess effectiveness. Again, this can include numeric results (percentage or proportion) or measures of association related knowledge, awareness and willingness to utilise and adhere to cervical cancer prevention services before and after integration or between integrated and non-integrated settings.*

*In addition, this review will also report qualitative data (where available) relating to the knowledge, awareness and willingness of AGYW living with HIV to utilise and adhere to cervical cancer prevention strategies following integration.”*

**Response to Reviewer 2**

**Comment 1:**

"Adolescent girls and young women (AGYW) face heightened risk of persistent HPV infection, a primary cause of cervical cancer." This statement is factually incorrect.

**Author’s response to comment 1:**

We thank the reviewer for picking up this incorrect statement and apologise for the mistake. This statement has been revised on page 2, line 26-29, which now reads:

*"Adolescent girls and young women (AGYW) living with HIV face heightened risk of persistent HPV infection, a primary cause of cervical cancer, making this population the ideal target for preventing cervical cancer before HPV exposure or disease progression."*

**Comment 2:**

Why is the review being limited to studies reported after 2006. Though I agree that HPV vaccination was not available before 2006, but the review also includes cervical cancer screening and management of precancerous lesions. This restriction of time shall lead to erroneous results in these two areas of cervical cancer prevention.

**Author’s response to comment 2:**

We thank the reviewer for this insightful comment on the timeline for this systematic review. However, we believe that the decision to limit this review to studies published after 2006 is justified by the transformative developments in cervical cancer prevention since this specified period. In addition to the introduction of licensed HPV vaccines, the period after 2006 has seen the 2016 United Nations General Assembly adoption of the Political Declaration on HIV and AIDS, which underscores the need for integrated services including prevention, screening and treatment of cervical cancer for persons living with HIV. Secondly, the global strategy to accelerate the elimination of cervical cancer as a public health problem published in 2020 gives further impetus for strengthening vaccination, screening and treatment of precancerous lesions as part of comprehensive prevention strategies particularly for those who are disproportionately affected like AGYW living with HIV.

There are several systematic reviews on cervical cancer screening which incorporate evidence published prior to 2006, and we do not wish to duplicate these efforts. Further to this, earlier studies prior to 2006 that were primarily focused on cytology-based screening methods no longer align with the WHO’s global guidelines. We therefore aim to synthesise evidence on pivotal developments in this space such as integration and utilisation of HPV-based screening, and updated approaches for managing precancerous lesions, such as the “screen, triage and treat” approach for females living with HIV, all of which will be pivotal additions to the existing body of knowledge. We trust that the Reviewer would agree that in focusing on evidence published post-2006 this review would provide timely, contextually relevant, high-quality evidence that aligns with current cervical cancer prevention guidelines.

This justification has now been included in our eligibility criteria on page 15, line 311-317 and now reads as follows:

*“3. Only studies published after 2006 will be considered, since the first commercially available prophylactic HPV vaccine was approved in that year. Further to this, earlier studies prior to 2006 that were primarily focused on cytology-based screening methods no longer align with the WHO’s global guidelines. We therefore aim to synthesise evidence on pivotal developments in this space such as integration and utilisation of HPV-based screening, and updated approaches for managing precancerous lesions.”*

**Comment 3:**

"HPV is estimated to infect approximately 291 million women globally, with a significantly higher prevalence among women under the age of 25 years". Though this statement is correct, the authors should also mention that majority of these HPV infections are known to clear on their own.

**Author’s response to comment 3:**

The Reviewer’s comment is well noted. This revision can be found in the “Background” section of the protocol on page 4, line 70-73, which reads as follows:

*“Persistent infection with the Human Papillomavirus (HPV) following sexual transmission is the primary sufficient cause of cervical cancer [2]. Furthermore, cervical cancer is the most common HPV-related disease [2]. Most HPV infections resolve spontaneously and do not cause symptomatic disease.”*

**Comment 4:**

"There are significant disparities in the prevalence of cervical cancer globally which highlights variations in accessibility..." This paragraph is not required and may be removed.

**Author’s response to comment 4:**

This paragraph has been removed.

**Comment 5:**

The sections on introduction of HPV vaccine, cervical cancer screening and treatment are very long and unnecessary. These need to be shortened extensively.

**Author’s response to comment 5:**

We thank the reviewer for this insight. These sections have been shortened. The revised version can be found on page 5-6, line 105-134, which now reads:

*“****Primary prevention***

*Prophylactic HPV vaccination has been regarded as the most effective long-term strategy for preventing HPV infection, cervical cancer and other cancers associated with HPV [5]. The first HPV vaccine was licensed in 2006 [7]. HPV vaccines are designed to prevent over 95% of HPV infections caused by the common high-risk HPV types 16 and 18 as well as some cross protection against other less common HPV types that can cause cervical cancer [1]. The WHO recommends the following schedule: a one or two-dose schedule for girls aged 9-14 years, a one or two-dose schedule for girls and women aged 15-20 years and two doses with a 6-month interval for women older than 21 years [6]. A minimum of 2 doses and when feasible 3-doses remain necessary for those known to be immunocompromised and/or HIV-infected [6]. The high prevalence of HPV infections in HIV positive females emphasizes how important it is to vaccinate this population. Furthermore, HPV vaccines are most effective if administered prior to HPV-exposure [7].*

*Educational interventions play a crucial role in creating awareness and imparting knowledge on the importance of cervical cancer prevention strategies and risks. In providing the necessary information females are empowered to make informed decisions about their health, seek HPV vaccination services and timely screening, as well as adopt proactive measures and ultimately reduce their risk of developing cervical cancer.*

***Secondary prevention***

*To prevent cervical cancer females can undergo various cervical screening tests to detect precancerous cells and thereafter receive timely and appropriate treatment to reduce the risk of progression to invasive cervical cancer. The conventional approach for screening involves cytology screening (conventional or liquid based), commonly known as Papanicoloau (Pap) test or pap smear [8]. Advances in screening strategies include visual inspection with acetic acid (VIA) and molecular tests such as high-risk HPV DNA testing [8].*

*In resource-limited settings, the predominant approach for treatment of cervical abnormalities is* *tissue ablation techniques such as cryotherapy or thermal ablation [9]. For women who are not eligible for ablation or in high income countries the main approach for treatment involves the* *excision of histologically confirmed cervical abnormalities [9].”*

**Comment 6:**

"According to WHO recommendations, females aged 9-20 years should receive one- or two-dose schedules and females 21 years and older should receive two-doses with a six- month interval". This statement is incorrect and in contradiction to the WHO Position Paper on HPV Vaccine, 2022. The reference provided for this statement is not accessible on the web. The authors need to check this information so that wrong information is not provided to the readers.

**Author’s response to comment 6:**

We thank the reviewer for picking up this discrepancy and apologise for the mistake. This statement has been revised with an appropriate reference provided, and can be found on page 5-6, line 110- 115, which now reads:

*“The WHO recommends the following schedule: a one or two-dose schedule for girls aged 9-14 years, a one or two-dose schedule for girls and women aged 15-20 years and two doses with a 6-month interval for women older than 21 years [6]. A minimum of 2 doses and when feasible 3-doses remain necessary for those known to be immunocompromised and/or HIV-infected [6].”*

**Comment 7:**

"Such integration can potentially improve access to cervical cancer screening, early detection, and pre-treatment services for AGYW living with HIV". Even in the WHO Guidelines, cervical cancer screening may begin at 21 years of age in women living with HIV. Hence, limiting this review to studies conducted in adolescent girls and young women would greatly limit the results reported thereof. For instance, the study of Mwanahamuntu et al reported cervical cancer screening in women living with HIV with mean age of 34 years.

**Author’s response to comment 7:**

We thank the Reviewer for this comment. We have amended the above referenced statement to reflect timely access to HPV vaccination for 9-14, 15-20, and ≥21-year-old AGYW, as well as initiation of cervical cancer screening at 25 years of age for AGYW living with HIV per WHO recommendations. The revised statement can now be found on page 8, line 160-162, which reads as follows:

*“**For AGYW living with HIV, such integration can potentially improve access to HPV vaccination, and timely initiation of cervical cancer screening, resulting in early detection and treatment.”*

**Comment 8:**

"Moreover, the search end dates of these reviews are outdated being more than six years old- and do not consider recent recommendations and advancements in the field." Which reviews are being referred to here? No references have been provided.

**Author’s response to comment 8:**

We thank the reviewer for pointing this out. We have now included appropriate reference to these previous reviews. The revision can be found on page 12, line 248-253, which now reads:

“*Previous studies have considered integration of these programs [12], although, more focus is placed on cervical cancer screening as a prevention strategy as opposed to a comprehensive approach that includes vaccination, education, and treatment of precancerous lesions. Moreover, the search end dates of previous reviews are outdated - being more than six years old - and do not consider recent recommendations and advancements in the field [12].”*

**Comment 9:**

For Objective 2, what is the definition of effectiveness of integrating cervical cancer prevention strategies into HIV care programs? Which parameters would be extracted from the included studies for this outcome?

**Author’s response to comment 9:**

We provide detailed descriptions of the parameters of interest where this is concerned as part of the Rationale, Objectives, and Outcomes of Interest sections. Under the “Outcomes of Interest” section on page 16-17, line 355-376, this now reads as follows:

*“2. We will also report on the effectiveness of integrating cervical cancer prevention strategies into existing HIV care programs. This will be assessed in terms of the following outcomes:*

*2.1 The uptake of cervical cancer prevention services (HPV vaccination, cervical cancer screening, treatment of precancerous lesions, and educational interventions) by AGYW living with HIV before vs after integration. Changes in measurable indicators related to the uptake of cervical cancer prevention strategies reported in included studies will be used to assess effectiveness. This can be numeric results (percentage or proportion), or measures of association related to uptake that have been reported before and after integration or those that compare uptake of services between integrated and non-integrated settings.*

*2.2The knowledge, awareness, and willingness of AGYW living with HIV to utilise and adhere to cervical cancer prevention strategies following integration. Changes in measurable indicators related to knowledge, awareness and willingness reported in included studies will be used to assess effectiveness. Again, this can include numeric results (percentage or proportion) or measures of association related knowledge, awareness and willingness to utilise and adhere to cervical cancer prevention services before and after integration or between integrated and non-integrated settings.*

*In addition, this review will also report qualitative data (where available) relating to the knowledge, awareness and willingness of AGYW living with HIV to utilise and adhere to cervical cancer prevention strategies following integration.”*

**Comment 10:**

How would knowledge, awareness, and willingness combined in the review? The reporting units of various studies could vary significantly.

**Author’s response to comment 10:**

This is addressed as part of the response to comment #9. We further describe how we intend to consolidate quantitative and qualitative data under the “Combining quantitative and qualitative data” section which can be found on page 24, line 533-540. Briefly, we will make use of guidelines provided by the Cochrane Handbook and Joanna Briggs Institute, adopting convergent integrated or convergent segregated approaches during our data analysis as appropriate.

**Comment 11:**

What would be the comparator for the various parameters related to integration of cervical cancer prevention strategies in HIV care programs?

**Author’s response to comment 11:**

Where studies are available that make use of before and after designs, we will explore the effectiveness of intervention versus no intervention or integration versus no integration.

**Comment 12:**

For Outcome 1, what would be the data extracted from the included studies? How would that be assessed in the review?

**Author’s response to comment 12:**

We have now elaborated on the anticipated outcomes of this review for better clarity. A revised version of outcome 1 can be found on page 16, line 339-353, which reads as follows:

“***Outcomes of interest***

1. *We will identify and describe types or models of integrated services utilised by AGYW living with HIV to access cervical cancer prevention services. Sigfrid et al, 2017 identify three models for integrating cervical cancer with HIV healthcare services [13]. The first model refers to within-clinic integration using internal staff, where the existing clinic structure and staff are used to incorporate a new set of services to complement services that are already provided [13]. In the second model, integration is achieved through co-location, where HIV and cervical cancer services are provided to the patient through coordination of care between different specialists or clinics within the same health care facility [13]. The third model involves complex programs of integration and coordination, including programs that integrated services by involving a range of different types of health workers (often from community health workers to clinical specialists) and facilities, and established systems to ensure clinical coordination and follow-up of patients [13]. Drawing on these three models, we will report on how cervical cancer prevention strategies are commonly integrated into HIV care services across the evidence base.”*

**Comment 13:**

For Outcome 2, there should be a comparator to assess the effectiveness against non-integration. In absence of a comparator, the results and conclusion would be less meaningful.

**Author’s response to comment 13:**

We thank the reviewer for this comment. As indicated, we will explore the effectiveness of intervention versus no intervention or integration versus no integration.
