# Supplementary material for: Effectiveness of integrating cervical cancer prevention strategies into HIV care programmes: A mixed-methods systematic review protocol
Source: PLoS One. 2025 May 19;20(5):e0306496. doi: 10.1371/journal.pone.0306496 (PMC12088019; doi:10.1371/journal.pone.0306496)
Supplement: Attachment — Submitted filename: Response to Reviewer.docx [file pone.0306496.s006.docx]

**Response to Reviewer 1**

**Comment 1:**

The rationale of including AGYW living with HIV is still not acceptable. The systematic review protocol aims to include all cervical cancer prevention strategies. When the WHO guidelines advocate cervical cancer screening in WLHIV starting at 25 years of age, how would the data align with the objective of the usefulness of cervical cancer screening in HIV care? The authors need to rethink the age group to be included. It would be better to have two age tiers**.**

**Author’s response to comment 1:**

We would like to thank the reviewer for this insightful concern regarding the age group for the participants in this review. While we acknowledge the WHO guidelines recommending cervical cancer screening in females living with HIV starting at the age of 25 years, we firmly believe in the merit this review would have if we were able to successfully demonstrate the effectiveness of screening in AGYW living with HIV. This would aid in facilitating timely identification of HPV infection before progression to cervical cancer.

As mentioned in the rationale AGYW living with HIV experience a higher risk of persistent infection and rapid progression to cervical precancerous lesions. Studies have shown that females living with HIV have a higher prevalence of high-risk HPV types even before the recommended age of 25 years [1]. For example, in South Africa HIV infection was found to be linked to persistence of HPV infection and a higher risk of Pap smear abnormalities in South African females aged 17–21 years [2]. Furthermore, cervical cancer rates begin to increase from 25-29 years [3]. Early screening in AGYW living with HIV could therefore serve as a preventive strategy in detecting and treating precancerous lesions before progression to cervical cancer in older age groups. If this review can demonstrate integration of the prevention strategies with HIV care to be effective among AGYW living with HIV this would mean that females in this population would make use of these integrated services and in turn potentially reduce the cervical cancer rates in females older than 25 years.

**Comment 2:**

The rationale of including studies post-2006 is also not acceptable. On the one hand, the authors claim that their systematic review shall have a global significance and on the other, they state that pre-2006, studies on cytology-based screening methods do not align with the WHO global guidelines. The authors need to revisit the WHO cervical cancer screening guidelines. The guidelines state that primary HPV-based screening is the standard. However, if this is not feasible, standardized cytology-based cervical cancer screening is acceptable. Hence, inclusion of only studies published after 2006 would bias the results highly and is not advocated. Duplication of earlier reviews is never an issue with a new systematic review and studies are not excluded solely on this basis.

**Author’s response to comment 2:**

We would like to thank the reviewer for this insightful comment on the timeline for this systematic review.

We have now revised the methods section for this protocol with the guidance provided which can be found on page 15, line 311-314, and reads as follows:

“*Initially, we will not place any time limitations on our search strategy to ensure that we comprehensively scope the evidence base for relevant literature. We do anticipate that any literature relating to HPV vaccination in our target population will be published from 2006 onwards in line with when the vaccine was first made available.”*

**Comment 3:**

In order to report on the effectiveness of integration of cervical cancer prevention strategies into HIV care programs, only studies with a before-and-after design should be included. This should be specifically stated.

**Author’s response to comment 3:**

We would like to thank the reviewer for this comment. We have included a statement in the protocol to highlight this point.

We have revised the outcome outlined for this review which can be found on page 16-17, line 350-362, which now reads:

2. *“We will also report on the effectiveness of integrating cervical cancer prevention strategies into existing HIV care programs. This will be assessed in terms of the following outcomes:*
   2.1 *The uptake of cervical cancer prevention services (HPV vaccination, cervical cancer screening, treatment of precancerous lesions, and educational interventions) by AGYW living with HIV before versus after integration. Changes in measurable indicators related to the uptake of cervical cancer prevention strategies reported in included studies will be used to assess effectiveness. This can be numeric results (percentage or proportion), or measures of association related to uptake that have been reported before and after integration or those that compare uptake of services between integrated and non-integrated settings. To assess this outcome studies that employ a before-and-after study design will be used.”*

**References:**

1. Burmeister CA, Khan SF, Schäfer G, Mbatani N, Adams T, Moodley J, et al. Cervical cancer therapies: Current challenges and future perspectives. Tumour Virus Res. 2022;13: 200238. doi:10.1016/j.tvr.2022.200238

2. Whitworth H, Changalucha J, Baisley K, Watson‐Jones D. Adolescent Health Series: HPV infection and vaccination in sub‐Saharan Africa: 10 years of research in Tanzanian female adolescents ‐ narrative review. Trop Med Int Health. 2021;26: 1345–1355. doi:10.1111/tmi.13660

3. Moscicki A-B, Perkins RB, Saville M, Brotherton JML. Should Cervical Cancer Screening be Performed Before the Age of 25 Years? J Low Genit Tract Dis. 2018;22: 348–351. doi:10.1097/LGT.0000000000000434
